# Evaluating long-term recurrence of cholesteatoma following primary surgery: an umbrella review of surgical techniques and patient factors

**DOI:** 10.1017/S0022215126105234

**Published:** 2026-08

**Authors:** Edi Handoko, Rizki Ekaputra Handoko, Ahmad Dian Wahyudi

**Affiliations:** Otorhinolaryngology-Head and Neck Surgery, Universitas Brawijaya, Indonesia

**Keywords:** cholesteatoma, endoscopy, middle ear, mastoidectomy, otolaryngology

## Abstract

**Objectives:**

This study aimed to synthesise existing systematic reviews to determine the true long-term recurrence rate of cholesteatoma following primary surgery and to evaluate the comparative effectiveness of surgical approaches (canal wall up vs canal wall down ) and adjunctive procedures on disease recidivism.

**Methods:**

A systematic search of PubMed, Scopus and OpenAlex identified 23 studies (predominantly systematic reviews and meta-analyses) encompassing 12,819 patients with an equal to or greater than 12 months’ follow-up and clearly defined recurrence, evaluating canal wall up, canal wall down, mastoid obliteration and endoscopic assistance.

**Results:**

Recurrence increased from 7 per cent at 12 months to 39 per cent at 15 years. Canal wall down showed lower recurrence than canal wall up. Mastoid obliteration (odds ratio, 0.33–0.45) and endoscopic techniques (risk ratio, 0.48–0.68) reduced recidivism and residual disease. Age and staged surgery increased risk, whereas planned second-look surgery was protective.

**Conclusion:**

Cholesteatoma recurrence is strongly time-dependent. Combining appropriate surgical techniques with mastoid obliteration, endoscopic assistance and long-term surveillance improves long-term disease control.

## Introduction

Management of cholesteatoma within the field of otology remains a significant clinical challenge due to the high risk of disease recidivism and the complexity of middle ear anatomical structures.^1^ The primary objective of primary surgery is the complete eradication of disease to prevent intracranial or extracranial complications, whilst simultaneously maintaining or restoring physiological ear functions.^1^ Despite various advancements in surgical techniques, recurrence rates remain the primary parameter for determining the long-term success of such procedures.^1^

A critical hurdle in evaluating the success of cholesteatoma surgery is the dependence of recurrence rates on follow-up duration.^1^ Short-term studies often underestimate the true disease burden, as recent evidence indicates that recurrence rates increase substantially over time, rising from 7 per cent at 12 months to reaching 39 per cent after 15 years.^1^ This phenomenon underscores that cholesteatoma requires standardised long-term surveillance to obtain an accurate clinical picture regarding the efficacy of interventions.^1^

The choice of primary surgical technique, particularly the comparison between canal wall up (CWU) and canal wall down (CWD), remains a subject of intense debate.^2^ Although CWD techniques are frequently associated with lower recurrence rates in adult populations, these results are often non-significant in paediatric-specific analyses.^1^^,^^3^ Beyond basic techniques, adjunctive approaches such as mastoid obliteration and the use of ear endoscopy have emerged as promising protective strategies.^1^^,^^4^ Mastoid obliteration is consistently reported to significantly reduce the risk of recurrence,^1^^,^^4^^,^^5^ whilst endoscopy enhances visualisation of hidden anatomical areas such as the sinus tympani, which is often a site of residual disease.^1^^,^^6^^,^^7^

Despite these innovations, current literature continues to exhibit substantial heterogeneity in recurrence definitions and outcome reporting methodology.^1^^,^^8^ Many studies fail to distinguish clearly between recurrent and residual disease and are limited by retrospective designs prone to selection bias.^1^^,^^8^ Furthermore, the identification of independent risk factors such as patient age, disease stage and the role of planned second-look surgery requires a more comprehensive synthesis of data based on the latest evidence.^1^ Therefore, this umbrella review and meta-analysis aims to comprehensively evaluate the long-term recurrence rates of cholesteatoma following primary surgery.^1^ This research synthesises evidence from multiple sources to analyse the impact of surgical techniques, adjunctive procedures and patient risk factors on long-term therapeutic success.^1^^,^^7^

## Material and methods

### Literature search strategy

**Figure 1.**
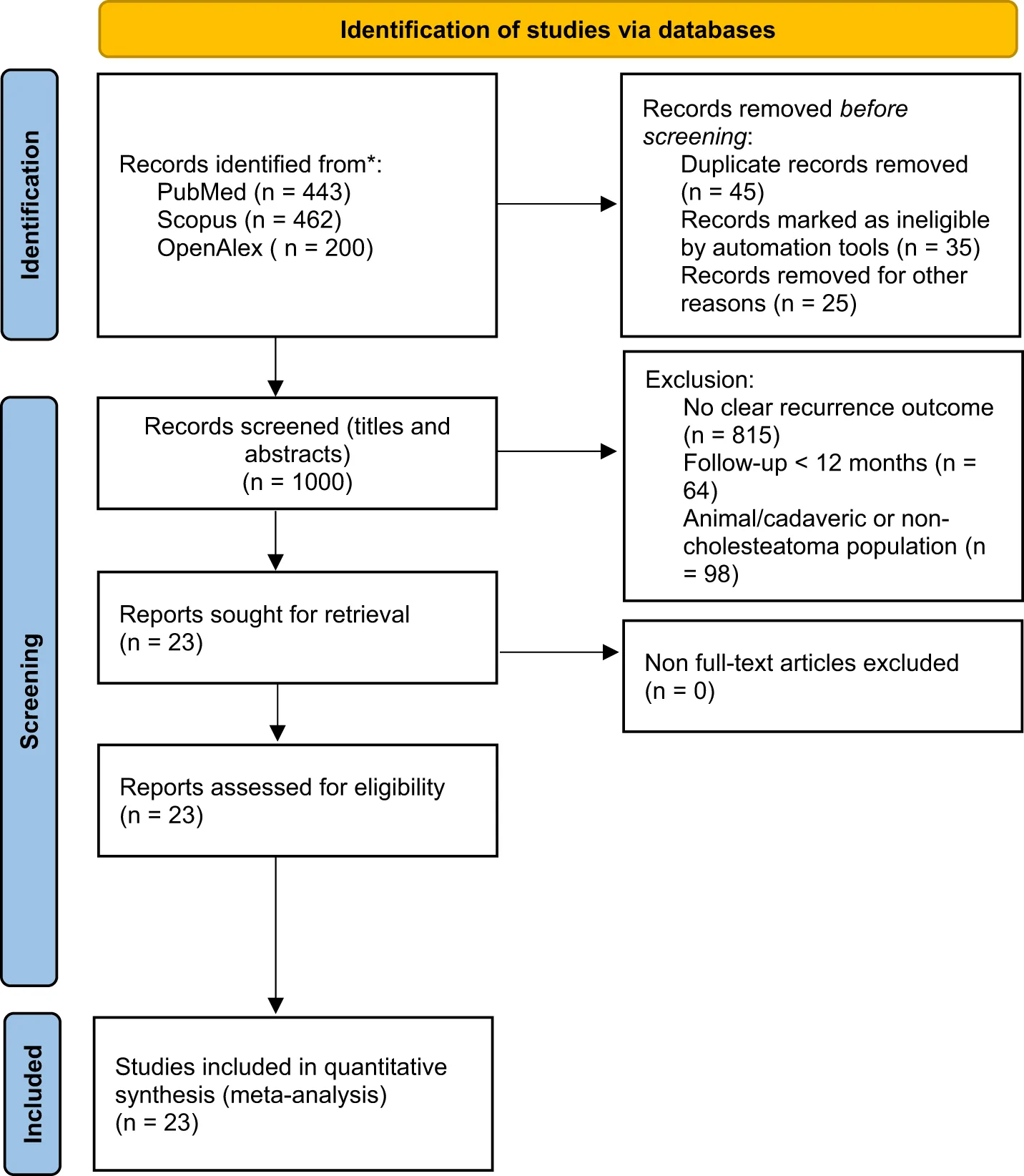
PRISMA flow diagram illustrating the literature search and study selection process. Figure 1 long description.

This umbrella review was conducted in accordance with the Preferred Reporting Items for Systematic Reviews and Meta-Analyses (PRISMA) guidelines (Figure 1). A comprehensive literature search was performed across major academic databases, including PubMed, Scopus and OpenAlex, encompassing over 138 million records. The search strategy combined terms related to cholesteatoma, recurrence, mastoidectomy, CWU, CWD, mastoid obliteration and endoscopic ear surgery and was limited to human studies with available full text. The initial electronic search retrieved the 1,000 most relevant records to the predefined clinical question on long-term cholesteatoma recurrence after primary surgery. This cap was applied post-search via the databases' automated sorting interfaces due to the vast size of the literature pools. “Relevance” was strictly defined by the databases' proprietary default algorithms, which prioritise keyword frequency and density in the titles and abstracts over publication date. Whilst this pragmatic cap ensured a focus on highly pertinent literature, it is explicitly acknowledged as a methodological limitation that may introduce selection bias. All records were exported into a reference management system, and duplicate entries were checked; as the search engine de-duplicated across sources, no further duplicate removal was required at this stage.

### Study selection and screening

Study selection proceeded in two sequential phases. In the first phase, titles and abstracts of all 1,000 records were screened against broad eligibility domains, including: (i) appropriate study design (predominantly systematic reviews and meta-analyses, randomised controlled trial, cohort study or case series with more than 10 patients); (ii) population with histologically or clinically confirmed cholesteatoma; (iii) reporting of recurrence or recidivism as an outcome; and (iv) minimum follow-up duration of 12 months. Records were excluded at this stage if they were case reports, small case series, narrative reviews without extractable data, animal or cadaveric studies or conference abstracts that lacked sufficient extractable data on recurrence outcomes or if they did not focus primarily on cholesteatoma surgery and recurrence. This process resulted in the exclusion of 977 records and the retention of 23 articles for full-text assessment.

In the second phase, the full texts of the remaining 23 articles were assessed in detail to confirm eligibility. Studies were included if they: (i) involved human participants with acquired cholesteatoma undergoing their first primary surgical intervention; (ii) provided a clear definition of recurrence and, where possible, distinguished it from residual disease; (iii) reported recurrence outcomes stratified by surgical technique, adjunctive procedures, or patient factors; and (iv) offered sufficient methodological detail to allow extraction of follow-up duration and key effect estimates. No additional full-text articles were excluded at this stage, resulting in a final sample of 23 studies included in the qualitative synthesis and quantitative meta-analysis.

### PICO framework

The review was structured based on the PICO (Population, Intervention, Comparison, Outcome) framework to ensure that the research question and data extraction remained focused and clinically meaningful. The components are summarised in Table 1 and include: patients of any age with acquired cholesteatoma undergoing primary surgical intervention (Population); primary surgical techniques such as CWU, CWD and endoscopic or microscopic approaches (Intervention); comparisons between different surgical techniques and adjunctive procedures such as mastoid obliteration (Comparison); and long-term recurrence rates as the primary outcome, with residual disease, patient-related risk factors and independent predictors of recidivism as secondary outcomes.

**Table 1.** PICO (Population, Intervention, Comparison, Outcome) framework Table 1 long description.

Component	Description
Population (P)	Patients of any age with acquired cholesteatoma undergoing primary surgical intervention.
Intervention (I)	Primary surgical techniques including canal wall up (CWU), canal wall down (CWD) and endoscopic or microscopic approaches.
Comparison (C)	Comparative analysis between different surgical techniques and the use of adjunctive procedures such as mastoid obliteration.
Outcome (O)	*Primary:* Long-term recurrence rates (up to 15 years). *Secondary:* Residual disease rates, impact of patient factors (age, stage) and independent predictors of recidivism.

### Risk of bias and study quality assessment

The methodological quality of the included studies was assessed independently by two reviewers using predefined criteria tailored to the study design. For systematic reviews and meta-analyses, quality was evaluated using the AMSTAR-2 (A MeaSurement Tool to Assess systematic Reviews) instrument, focusing on the clarity of the research question, transparency of inclusion criteria and completeness of the search strategy. Primary observational studies were assessed using the Newcastle–Ottawa Scale for potential selection bias, completeness of follow-up and control of confounding factors. Based on these assessments, the overall quality of each source was classified as “Good”, “Fair” or “Limited”. A summary of the quality assessment for the key included sources is presented in Table 2.

**Table 2.** Quality assessment and characteristics of all included sources (*n* = 23) Table 2 long description.

Reference	Study type	Sample size (patients/studies)	Primary focus	Quality level*
Saqr Massoud *et al.* (2025)	SR/meta-regression	12,819 patients (84 studies)	Determinants of recurrence	Good
J. Salem *et al.* (2022)	SR/meta-analysis	2,379 patients	CWD-MO vs CWD vs CWU	Good
H. van der Toom *et al.* (2018)	Systematic review	1,534 patients	CWU vs CWD with obliteration	Good
Paola Solis-Pazmino *et al.* (2023)	SR/meta-analysis	1,333 patients	CWU vs CWD (paediatric)	Good
Bo Li *et al.* (2020)	SR/meta-analysis	Not specified	Endoscopic vs microscopic	Good
S. Nair *et al.* (2021)	SR/meta-analysis	13 studies	Endoscopic vs microscopic	Good
R. B. Giffoni *et al.* (2025)	SR/meta-analysis	1,134 cases	Endoscopic-guided surgery	Good
Francesca Viberti *et al.* (2024)	SR/meta-analysis	2,882 obliterated ears	Graft materials	Good
N. Mor *et al.* (2013)	Systematic review	43 articles	Outcome measure variability	Fair
L. Presutti *et al.* (2014)	Systematic review	515 patients	Endoscopic ear surgery	Good
Razan A. Basonbul *et al.* (2020)	SR/meta-analysis	14 articles	Endoscopic (paediatric)	Good
B. Verma *et al.* (2020)	Systematic review	1,685 patients	Endoscopic adjunct	Good
K. Lau *et al.* (2020)	Systematic review	536 participants	Laser technology	Good
A. Moffa *et al.* (2021)	Systematic review	607 patients	Mesna application	Good
Mónica Rueda Vega *et al.* (2024)	Cohort/review	94 cases	Petrous bone cholesteatoma	Fair
K. Illés *et al.* (2022)	SR/meta-analysis	2,077 operations	Mastoid obliteration vs CWU	Good
K. Illés *et al.* (2023)	Correspondence	Not specified	Subgroup analysis	Limited
L. La Via *et al.* (2023)	Correspondence	1,847 patients	Mastoid obliteration vs CWU	Limited
J. Mulder *et al.* (2016)	Systematic review	Not specified	CWU vs CWD	Good
H. Ching *et al.* (2017)	SR/meta-analysis	45 patients	Paediatric TM cholesteatoma	Fair
Flora Yan *et al.* (2020)	SR/meta-analysis	377 patients	Subtotal petrosectomy	Good
P. Morris *et al.* (2012)	Systematic review	51 studies	CSOM treatments	Fair
A. Mustafa *et al.* (2008)	Retrospective cohort	223 patients	CWU vs CWD	Fair

### Data synthesis

As an umbrella review, data synthesis was designed to integrate findings across previously published systematic reviews and meta-analyses, capturing heterogeneous populations, surgical techniques and follow-up durations in a clinically interpretable manner. A narrative synthesis was employed to reconcile aggregated results from these reviews—which encompassed numerous primary studies with differing designs and outcome definitions—with particular attention to patterns in time-dependent recurrence and the impact of adjunctive procedures. By extracting outcome data directly from the included reviews, this umbrella review synthesises evidence derived from a much larger pool of primary literature. Importantly, we did not reclassify recurrence outcomes at the primary study level; rather, we accepted each included review’s own definition of recurrence. Consequently, it must be acknowledged that the pooled estimates presented in this umbrella review may conflate residual and recurrent disease, reflecting the inherent limitations of the underlying literature. In addition, data from multivariable meta-regressions were analysed to identify independent predictors of recurrence, specifically adjusting for age, surgical staging and the role of planned second-look procedures. Findings were categorised by surgical technique (CWU vs. CWD), adjunctive procedures (mastoid obliteration and endoscopic assistance) and patient-specific risk factors, thereby allowing a structured interpretation of how surgical and patient-related variables influence long-term cholesteatoma control.

## Results

### Article search results

A comprehensive literature search was conducted using three electronic databases: PubMed, Scopus and OpenAlex. A total of 1,000 records were identified. After the removal of duplicate records and other ineligible records prior to screening, the remaining articles were screened based on titles and abstracts. During the title and abstract screening stage, 977 records were excluded for the following reasons: lack of clear recurrence outcomes (*n* = 815), follow-up duration of less than 12 months (*n* = 64) and studies involving animal or cadaveric models or non-cholesteatoma populations (*n* = 98).

As a result, 23 full-text articles were sought for retrieval and subsequently assessed for eligibility. All retrieved full-text articles met the inclusion criteria, and no studies were excluded at this stage due to unavailable or incomplete full texts. Finally, 23 studies were included in the qualitative synthesis as part of this umbrella review. These sources provided a robust evidence base encompassing over 12,800 patients. The included studies comprised predominantly systematic reviews and meta-analyses, with several primary studies contributing patient-level data. Demographic analysis of the pooled population across these reviews revealed a distribution of 4,642 males and 3,185 females. Follow-up durations across the included evidence were extensive, ranging from 6 to 300 months, which allowed for a detailed analysis of long-term outcomes. The key characteristics and recurrence outcomes of all 23 included sources are summarised in Table 3.

**Table 3.** Summary of characteristics and outcomes for all included sources (*n* = 23) Table 3 long description.

Reference	Population focus	Key finding/recurrence outcome	Significant predictors
Massoud (2025)^1^	Mixed (n=12,819)	7% (1 yr) to 39% (15 yr) recurrence	Age, staged surgery, second-look
Mustafa (2008)^2^	Adults/paediatric	CWU vs CWD outcomes in 223 cases	Surgical technique
Solis-Pazmino (2023)^3^	Paediatric	No sig. difference between CWU and CWD	Paediatric age
Salem (2022)^4^	Mixed	CWD-MO protective (OR 0.330)	Mastoid obliteration
Illés (2023)^5^	Mixed	MO reduced recidivism (OR 0.45)	Mastoid obliteration
Li (2020)^6^	Mixed	Endoscopy reduced residual (RR 0.68)	Endoscopic assistance
Nair (2021)^7^	Mixed	Endoscopic vs microscopic comparison	Surgical approach
Mor (2013)^8^	Not specified	Only 31/84 studies defined recurrence	Definition heterogeneity
Mulder (2016)^9^	Acquired	6% recurrence in CWU vs CWD	Surgical technique
Viberti (2024)^10^	Obliterated ears	Heterologous (3.4%) vs autologous (5.2%)	Graft material
van der Toom (2018)^11^	Mixed	CWU+MO recurrence rate of 0.28%	Mastoid obliteration
Presutti (2014)^12^	Mixed	9.3% recidivism in endoscopic surgery	Endoscopic visualisation
Giffoni (2025)^13^	Mixed	Reduced residual risk (RR 0.65)	Endoscopic-guided surgery
Basonbul (2020)^14^	Paediatric	Endoscopic RR 0.48 for residual disease	Endoscopic visualisation
Verma (2020)^15^	Mixed	15.82% residual detection via endoscopy	Endoscopic adjunct
Lau (2021)^16^	Mixed	0-33% recurrence range with lasers	Technical innovation
La Via (2023)^17^	Mixed	TSA confirms MO effectiveness (*n* = 1,847)	Statistical robustness
Illés (2023) (Reply)^18^	Mixed	Confirmed robustness of MO benefits	Mastoid obliteration
Moffa (2021)^19^	Acquired	Improved recurrence with Mesna application	Chemical adjunct
Ching (2017)^20^	Paediatric	2% recurrence; size correlates with age	Tumour size/age
Rueda Vega (2024)^21^	Petrous bone	8.51% recurrence in complex PBC cases	Endoscopic assistance
Yan (2020)^22^	Mixed	9.3% recurrence in subtotal petrosectomy	Surgical extent
Morris (2012)^23^	Mixed	Systematic review of CSOM treatments	Clinical management

### Risk of bias and quality assessment

The methodological quality of the included studies demonstrated substantial heterogeneity, particularly regarding the formal definition of recurrence outcomes. To clarify the scope of the synthesised data, while this umbrella review directly analysed 23 sources, these sources themselves extracted data from numerous primary studies. For instance, in the largest included meta-analysis (Massoud *et al*.), which aggregated data from 84 primary studies, only 31 of those underlying studies explicitly provided a clear definition of what constituted recurrent cholesteatoma. Furthermore, most studies failed to distinguish clearly between recurrent and residual disease. The predominance of retrospective designs across the literature introduced a potential for selection bias, which was acknowledged as a factor that may limit causal inference. Despite this, the inclusion of high-quality systematic reviews and the use of multivariable meta-regression in recent evidence provided a robust framework for identifying independent predictors of disease recidivism.

### Synthesis of recurrence outcomes and surgical techniques

Recurrence rates demonstrated a profound time-dependent increase, indicating that short-term follow-up leads to a significant underestimation of the true disease burden.^1^ Pooled data indicated a recurrence rate of 7 per cent at 12 months, which rose to 18 per cent at 5 years and peaked at 39 per cent at 15 years.^1^ Regarding surgical approaches, CWD techniques were generally associated with lower recurrence rates (7 per cent) compared to CWU techniques (16 per cent) in mixed adult and paediatric populations.^1^^,^^4^^,^^9^ However, this superiority was not observed in paediatric-specific analyses, where no significant difference between the two techniques was identified (RR 1.50; 95 per cent CI 0.94–2.40).^3^ This discrepancy may be attributed to the higher baseline recurrence risk in children, which was 13 per cent compared to 10 per cent in adults.^1^

### Adjunctive procedures and independent predictors

The use of adjunctive procedures significantly influenced long-term success rates. Mastoid obliteration consistently emerged as a potent protective factor across multiple meta-analyses, with odds ratios (ORs) ranging from 0.33 to 0.45.^4^^,^^5^^,^^10^ A specific systematic review (van der Toom *et al.*) reported an exceptionally low recurrence rate of 0.28 per cent when CWU was combined with mastoid obliteration; however, this represents a single-review estimate rather than a broadly generalisable outcome.^11^

Endoscopic approaches also demonstrated a significant reduction in residual disease compared to microscopic surgery, with risk ratios ranging from 0.48 to 0.68.^6^^,^^7^^,^^12^^–^^14^ Intra-operative endoscopy identified residual disease in hidden areas, such as the sinus tympani, in 15.82 per cent of cases after microscopic clearance was completed.^14^^,^^15^ Laser technology in cholesteatoma surgery exhibits a wide range of reported recurrence rates (0–33 per cent) with a safe hearing profile across included studies.^16^ Multivariable meta-regression identified three independent predictors of recurrence: patient age, staged surgery (risk-increasing) and planned second-look surgery (protective).^1^

## Discussion

This umbrella review of 23 sources demonstrates that cholesteatoma recurrence is a highly time-dependent phenomenon, with rates increasing nearly six-fold between one and 15 years of follow-up.^1^ Our findings underscore a critical paradigm shift in otological surveillance: short-term studies, which typically report outcomes within the first 12 to 24 months, significantly underestimate the true disease burden.^1^ The identification of a 39 per cent recurrence rate at 15 years, compared to only 7 per cent at 12 months,^1^ suggests that cholesteatoma remains a life-long risk for the patient. Consequently, this necessitates standardised, long-term follow-up protocols that extend well beyond the traditional five-year window to obtain an accurate clinical assessment of surgical success.^1^

The debate regarding CWU versus CWD techniques remains central to surgical selection. While our pooled analysis of mixed populations identified a significant advantage for CWD in reducing recurrence,^1^^,^^4^^,^^9^ typically reported at 7 per cent compared to 16 per cent for CWU,^1^ this benefit was not mirrored in paediatric-specific cohorts.^3^ The equivalent outcomes observed between techniques in children (RR 1.50; 95 per cent CI 0.94–2.40).^3^ may reflect the inherently more aggressive nature of paediatric cholesteatoma, where higher baseline recurrence rates of approximately 13 per cent compared to 10 per cent in adults potentially attenuate the relative technical advantages of CWD.^1^ This discrepancy likely stems from the inherently more aggressive biological nature of paediatric cholesteatoma, often characterised by higher inflammatory activity and poorer Eustachian tube function, which may attenuate the technical advantages of the CWD approach. A primary retrospective cohort study of 223 patients reported similar findings, with no substantial difference in outcomes between CWU and CWD approaches when controlling for disease characteristics.^2^ Furthermore, the choice between CWU and CWD in clinical practice is rarely random: surgeons may preferentially select CWD for more extensive disease, introducing “confounding by indication” bias that observational studies cannot fully address without randomised controlled trials.^1^ This selection bias potentially masks the true effectiveness of CWD by applying it to higher-risk populations.

Beyond surgical technique selection, disease-specific characteristics significantly influence recurrence risk. Acquired cholesteatoma demonstrates substantially higher recurrence rates (12 per cent) compared to congenital disease (7 per cent),^1^ whilst disease staging reveals a progressive increase in recurrence from Stage I (4 per cent) to Stage IV (18 per cent).^1^ These patient-level factors underscore the importance of risk stratification in surgical planning and highlight the need for multivariable analyses that adjust for baseline disease severity when comparing surgical approaches.

Mastoid obliteration emerged as one of the most consistently effective protective strategies identified in this review.^4^^,^^5^^,^^10^^,^^11^^,^^17^ The robust reduction in recidivism (OR 0.33–0.45),^4^^,^^5^^,^^10^ confirmed by trial sequential analysis with adequate information size (*n* = 1,847) establishing statistical robustness,^17^^,^^18^ supports the wider adoption of cavity elimination techniques. Interestingly, one systematic review noted that the synergy of maintaining the posterior canal wall anatomy (CWU) while eliminating the mastoid volume through obliteration yielded a reported recurrence rate as low as 0.28 per cent.^11^ Although this highlights the potential of the “obliteration effect,” it should be interpreted cautiously as it derives from a specific subset of studies rather than a universal pooled estimate.^11^ This suggests that the “obliteration effect” functions multifactorially: by reducing the mastoid cavity size requiring surveillance, facilitating posterior canal wall reconstruction and creating a physiological environment less conducive to retraction pocket formation and epithelial regrowth.^10^^,^^11^ The choice of obliteration material also appears clinically relevant, with heterologous materials demonstrating lower recurrent and residual disease rates (3.4 per cent) compared to autologous materials (5.2 per cent),^10^ albeit with slightly higher rates of otorrhoea (5.2 per cent vs. 4.9 per cent) and tympanic membrane perforation (4.3 per cent vs. 2.5 per cent).^10^

The integration of endoscopic assistance represents another significant advancement in reducing disease burden.^6^^,^^7^^,^^12^^,^^13^^,^^15^ Our analysis confirms that endoscopy significantly lowers the risk of residual disease by improving the visualisation of anatomically hidden areas, such as the sinus tympani and facial recess.^6^^,^^15^ The intra-operative detection of residual cholesteatoma in 15.82 per cent of cases after microscopic clearance was deemed complete.^14^^,^^15^ highlights that endoscopy should be viewed as an essential additive tool rather than a replacement for the operating microscope. The benefit of endoscopy appears attributable to enhanced visualisation rather than fundamentally different surgical principles,^15^ suggesting that a combined microscopic-endoscopic approach may represent optimal practice. By specifically targeting residual epithelial pearls that would otherwise go undetected, endoscopic techniques directly address one of the primary drivers of early surgical failure.^6^

Novel adjunctive techniques have also demonstrated promising results in reducing recurrence burden. In addition to laser technology,^16^ sodium 2-mercaptoethanesulfonate (Mesna) has shown potential as a chemical adjunct during cholesteatoma surgery, with all included studies demonstrating improvement in recurrence and residual disease rates without associated sensorineural hearing loss.^19^ These emerging technologies warrant further investigation in comparative effectiveness studies to establish their role in adjunctive surgical management.

A particularly intriguing finding from multivariable meta-regression is the paradoxical relationship between staged surgery and planned second-look procedures.^1^ Staged surgery (defined as two or more planned operations) was identified as a risk-increasing factor, whilst planned second-look surgery emerged as an independent protective factor.^1^ This apparent contradiction likely reflects confounding by indication: staged procedures may be preferentially selected for more extensive or complex disease where complete single-stage clearance is deemed unfeasible, thereby increasing baseline recurrence risk. Conversely, planned second-look surgery represents proactive surveillance in cases where complete disease eradication is uncertain, enabling early detection and management of residual disease before it progresses to frank recurrence. This distinction has important clinical implications, suggesting that protocolised second-look procedures should be considered in high-risk cases rather than as an indicator of surgical inadequacy.

Special consideration should be given to paediatric tympanic membrane cholesteatoma, a distinct clinical entity characterised by localised retraction pockets confined to the pars tensa. Although representing only a small proportion of paediatric cholesteatoma cases, this condition demonstrates notably lower recurrence rates (2 per cent) with disease size correlating significantly with patient age.^20^ Understanding the natural history and risk factors of this subtype may inform surveillance strategies and guide conservative management decisions in selected cases.

The role of extended surgical approaches in advanced or recurrent disease also merits consideration. Endoscopic assistance has proven valuable in complex cases such as petrous bone cholesteatoma.^21^ Furthermore, subtotal petrosectomy and cochlear implantation, whilst employed in specific scenarios involving extensive disease with concomitant hearing loss, demonstrate variable outcomes with reported recurrence rates ranging from 4.3 per cent to 17.1 per cent depending on patient selection and disease characteristics.^22^ These approaches represent important options in the surgical armamentarium for challenging cases where standard mastoidectomy techniques may be inadequate.

Despite these insights, several methodological limitations must be acknowledged. As noted in the large-scale analysis by Massoud *et al*., only 31 of 84 underlying primary studies explicitly defined the outcome. Furthermore, the distinction between recurrent cholesteatoma (reformation of a retraction pocket in the tympanic membrane) and residual disease (incomplete removal presenting as an epithelial pearl) was not consistently maintained across studies,^8^ potentially conflating two biologically and surgically distinct entities. The predominance of retrospective designs introduces selection bias that limits causal inference, particularly regarding the comparative effectiveness of surgical techniques.^1^ Additional limitations include the variable application of imaging modalities for recurrence detection, with only 12 per cent of studies presenting results as Kaplan–Meier disease-free curves,^8^ and the incomplete reporting of follow-up duration across studies. The broader literature on chronic suppurative otitis media management, whilst including cholesteatoma as one clinical manifestation, often lacks the disease-specific detail necessary for robust comparative analysis.^23^ Future research should prioritise the adoption of standardised reporting frameworks, such as the EAONO/JOS Joint Consensus Statements, to ensure methodological consistency and facilitate meaningful cross-study synthesis. Additionally, adequately powered randomised controlled trials are needed to definitively establish the causal effects of mastoid obliteration and endoscopic techniques whilst controlling for disease severity and other confounding variables.

**What is already known on this topic**

- Cholesteatoma surgery carries a substantial risk of long-term recurrence, which is often underestimated in studies with short follow-up.
- The relative benefits of canal wall up versus canal wall down techniques, mastoid obliteration and endoscopic assistance remain uncertain because of heterogeneous study designs and outcome definitions.

**What this paper adds**

- This umbrella review of 23 studies (12,819 patients) shows that cholesteatoma recurrence is strongly time-dependent, rising from 7 per cent at 12 months to 39 per cent at 15 years, underscoring the need for prolonged post-operative surveillance
- Mastoid obliteration and endoscopic assistance consistently reduce recidivism and residual disease, while age, staged surgery and planned second-look procedures emerge as key independent predictors of long-term recurrence
- The surgical advantage of canal wall down (CWD) techniques in reducing recurrence typically seen in mixed or adult populations is attenuated in paediatric cohorts, likely reflecting the inherently more aggressive biology and higher baseline recurrence risk of childhood cholesteatoma

## Conclusion

Long-term cholesteatoma recurrence is profoundly time-dependent, increasing from 7 per cent at 12 months to 39 per cent at 15 years, necessitating extended surveillance protocols beyond the traditional five-year window. While CWD techniques demonstrate lower recurrence in adult populations (7 per cent vs. 16 per cent), this advantage does not translate to paediatric cohorts, emphasising the need for individualised surgical selection based on patient age, disease extent and institutional expertise.

Mastoid obliteration, particularly when combined with CWU procedures, and endoscopic assistance represent the most-effective protective strategies. Evidence suggests that the synergistic combination of CWU with mastoid obliteration may yield exceptionally low recurrence rates in select cohorts, whilst endoscopic techniques successfully identify residual disease in approximately 15.82 per cent of cases after microscopic clearance. Future evidence requires standardised recurrence definitions, long-term follow-up protocols with structured imaging and randomised controlled trials to establish causal relationships whilst controlling for disease severity.

## Figure 1 Long description

The flowchart illustrates the identification and selection process of studies via databases for a meta-analysis. It begins with the identification phase, where records are identified from PubMed (443), Scopus (462) and OpenAlex (200), totaling 1,105 records. Before screening, 45 duplicate records, 35 ineligible records by automation tools and 25 records for other reasons are removed. This results in 1,000 records screened by titles and abstracts. During the screening phase, exclusions are made for 815 records with no clear recurrence outcome, 64 with follow-up less than 12 months and 98 involving animal/cadaveric or non-cholesteatoma populations. This leads to 23 reports sought for retrieval, all of which are assessed for eligibility. No non-full-text articles are excluded. Finally, 23 studies are included in the quantitative synthesis (meta-analysis).

Navigate back to Figure 1.

## Table 1 Long description

PICO framework defining a surgical research question for acquired cholesteatoma. Population includes patients of any age undergoing first-time surgery for acquired cholesteatoma. Interventions are primary surgical techniques, including canal wall up, canal wall down, and endoscopic or microscopic approaches. Comparisons examine differences among these techniques and the added use of adjunctive procedures such as mastoid obliteration. Outcomes prioritize long-term recurrence rates with follow-up extending to 15 years, with secondary outcomes including residual disease rates. The table also notes evaluation of patient factors such as age and disease stage, and identification of independent predictors of recidivism. No numerical results are provided, so it outlines what will be measured and compared rather than reporting findings.

Navigate back to Table 1.

## Table 2 Long description

Quality ratings and study characteristics are listed for 23 included sources, giving each reference’s study type, sample size, primary focus, and quality level. Most entries are systematic reviews or systematic reviews with meta-analysis, and most are rated Good quality. Sample sizes vary widely, from small clinical series such as 45 patients to large evidence syntheses such as 12,819 patients across 84 studies; several reviews and correspondence pieces do not report sample size. Common topics include comparisons of canal wall up versus canal wall down surgery, mastoid obliteration, and endoscopic versus microscopic approaches, with additional focuses such as graft materials, laser technology, Mesna use, petrous bone cholesteatoma, and chronic suppurative otitis media treatments. Fair-quality sources appear in several areas, including outcome measure variability, petrous bone cholesteatoma, paediatric tympanic membrane cholesteatoma, chronic suppurative otitis media treatments, and a retrospective cohort comparing canal wall techniques. Limited-quality items are correspondence pieces related to subgroup analysis and mastoid obliteration comparisons. Interpretation should note that evidence types are mixed and some entries lack sample size details, which can affect how directly studies can be compared.

Navigate back to Table 2.

## Table 3 Long description

Study characteristics and recurrence or recidivism outcomes are summarized across 23 sources, listing each study’s population focus, key recurrence finding, and reported significant predictors. Reported recurrence varies widely, from about 0.28 percent in canal wall up with mastoid obliteration to as high as 39 percent by 15 years in a large mixed cohort, highlighting strong dependence on follow-up duration and case mix. Several sources report mastoid obliteration as protective, including reduced odds or risk of recidivism and very low recurrence rates in combined approaches. Multiple studies compare endoscopic and microscopic techniques, with several reporting lower residual disease risk or improved detection when endoscopy is used as an adjunct or guidance. Comparisons of canal wall up versus canal wall down approaches show mixed findings, including studies reporting differences and others finding no significant difference, particularly in paediatric cohorts. Additional factors noted as predictors include age, staged surgery and second-look procedures, graft material in obliterated ears, tumour size in paediatric cases, and surgical extent in complex petrous bone or subtotal petrosectomy cases. One review notes that many studies do not clearly define recurrence, so differences in definitions and reporting may limit direct comparison across sources.

Navigate back to Table 3.
